# Supplementary material for: Biological and Immunological Characterization of a Functional L-HN Derivative of Botulinum Neurotoxin Serotype F
Source: Toxins (Basel). 2023 Mar 6;15(3):200. doi: 10.3390/toxins15030200 (PMC10056376; doi:10.3390/toxins15030200)
Supplement: Supplementary file 1 [file toxins-15-00200-s001.zip › toxins-2232107-supplementary.pdf]

Supplemental Materials

Biological and Immunological Characterization of a Functional L-HN Derivative of Botulinum Neurotoxin Serotype F

Table S1. Basic information for the BoNT/F functional molecules used in the present study.

Functional fragment	Amino acid sequence	Fragment size	Protein molecular weight	Fragment feature
FL	1-436	1308 bp	50 kDa	Light chain enzyme active region
FHN	437-857	1263 bp	50 kDa	Transmembrane region
FL-HN	1-857	2574 bp	100 kDa	Light chain enzyme - Transmembrane region
FmL-HN	1-857	2574 bp	100 kDa	Mutated Light chain enzyme - Transmembrane region
FsL-HN	244-627	1152 bp	43 kDa	Small Light chain enzyme-Transmembrane region
FH	537-1278	2226 bp	82 kDa	Heavy chain
FHc	858-1315	1374 bp	49 kDa	Receptor binding region
FHc-C	1016-1278	609 bp	25 kDa	C-terminal of receptor binding region
FHc-N	858-1075	654 bp	25 kDa	N-terminal of receptor binding region
rVAMP2	30-92	189 bp	8 kDa	Rat substrate protein of BoNT/F

Notes：FH, botulinum neurotoxin serotype F (BoNT/F) heavy chain; FHc, BoNT/F heavy chain receptor binding region, FHc-C, C-terminus of BoNT/F heavy chain receptor binding region; FHc-N, N-terminus of BoNT/F heavy chain receptor binding region; FHN, BoNT/F heavy chain transmembrane region; FsL-HN, BoNT/F small light chain enzyme-transmembrane region; FL-HN, BoNT/F light chain enzyme-transmembrane region; FmL-HN, BoNT/F mutated light chain enzyme-transmembrane region; rVAMP2 (aa30-92) was fused to thioredoxin (Trx) to produce a fusion protein Trx-rVAMP2; Trx; thioredoxin; rVAMP2, Rat substrate protein of BoNT/F. These DNA sequences encoding the BoNT/F (Langeland strain, accession:X81714, amino acids 1 to 1278) functional fragments and the amino acids 30-92 sequence of rVAMP2 were synthesized by Beijing Huada Gene Co. , Ltd. in this study. The genes encoding each molecule (FL, FHN, FL HN, FsL-HN, FH, FHc, FHc-N, FHc-C, FmL-HN, and rVAMP2) were inserted into the prokaryotic expression vector pTIG-Trx to production of recombinant proteins in Escherichia coli BL21 (DE3).

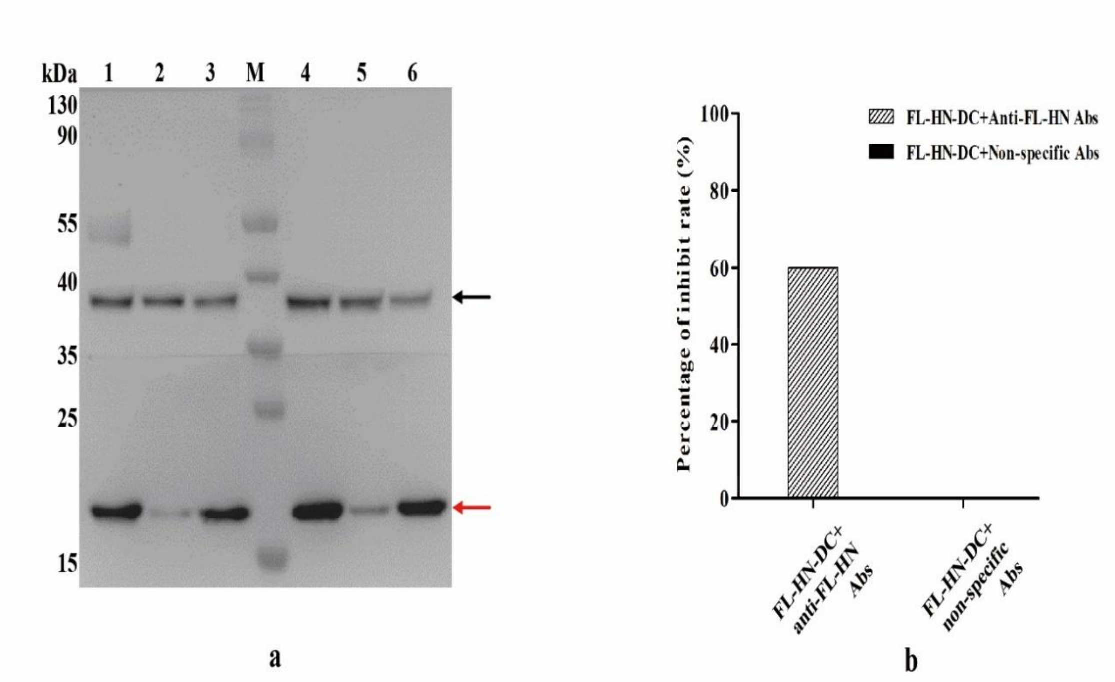

Figure S1. FL-HN-DC activity inhibition assay at the cellular level. a, Enzyme activity inhibition assay of FL-HN-DC was determined by western blotting in Neuro-2a cell (one of three representative experiments). The black and red arrows indicate the uncleaved GAPDH and VAMP2, respectively. Lane 1, Cell + FL-HN-DC (100 nM) + specific anti-FL-HN serum antibodies (anti-FL-HN Abs, 1:10), lane 2, Cell + FL-HN-DC (100 nM)+ non-specific sera antibodies (non-specific Abs, 1:10), lane 3, Cell+ FL-HN-DC (100 nM)+ anti-FL-HN Abs (another sample), lane 4, Cell + FL, lane 5, Cell + FL-HN-DC (100 nM); lane 6, only cell without FL-HN-DC (cell); b, Quantification of inhibition of FL-HN-DC activity in Neuro-2a cell by specific anti-FL-HN antibodies. Results were obtained by densitometry analysis and plotted. Densitometric analysis was performed using Image J software and bar chart was performed using GraphPad prism 8.
